# Keeping the Fire House Running: A Proposed Approach to Mitigate Spread of COVID-19 Among Public Safety Personnel

**DOI:** 10.5811/westjem.2020.3.47298

**Published:** 2020-03-15

**Authors:** Robert J. Katzer

**Affiliations:** University of California, Irvine, Department of Emergency Medicine, Irvine, California

## BACKGROUND

Originating within the city of Wuhan, Hubei Province, China, in December 2019 and January 2020, the disease COVID-19 has spread widely throughout the world.^1^–^3^ On March 11, 2020, the World Health Organization (WHO) classified COVID-19 as a pandemic. COVID-19 is caused by the coronavirus SARS-CoV-2, and is believed to have originated from bats.^4^–^6^ Its overall case fatality rate has been reported between 1% and 3.8%, although this number is likely to evolve as broader testing becomes available for less severe cases.^1^,^6^,^7^ The WHO-China Joint Mission on Coronavirus determined that 75–85% of case clusters in China occurred within families, presumably in the household.^4^

Across the world, efforts are underway to contain the spread and mitigate the impact of COVID-19. These include social distancing efforts such as working from home and meeting via teleconferences.^8^ The nature of public safety both necessitates that first-responder personnel be present at the station and requires vigilance to keep them healthy to provide essential services to the community. As a result, the fire station represents a front line in the COVID-19 mitigation efforts. The impact on fire department staffing was demonstrated when 25 of 111 employees of the City of Kirkland, Washington, Fire Department were placed under quarantine after responding to calls at a single, skilled nursing facility later found to have a COVID-19 case cluster.^9^

Given the annual presence of the influenza virus, comparing influenza to COVID-19 provides some basis to evaluate the threat. Where COVID-19’s estimated case fatality rate is between 1% and 3.8%, the United States Centers for Disease Control and Prevention (CDC) estimates influenza’s overall case fatality rate at around 0.1%.^10^ Furthermore, it is estimated that each case of COVID-19 will infect an average of between 2 and 2.5 other people.^6^ This ratio is referred to as the reproductive number. A literature review found the reproductive number of seasonal influenza to be lower, at 1.19 and 1.37.^11^

The emergence of a contagious disease with a higher reproductive number and 10–40 times more lethal than seasonal influenza should concern all of us. Understanding how the disease is spread is paramount to mitigate its spread. As with many coronaviruses, evidence indicates that COVID-19 is spread via respiratory droplets.^4^,^8^,^12^ COVID-19 can travel directly from an infected person to a nearby person after coughing or sneezing. It has been collected on the surfaces of a patient’s hospital room after hours to days.^13^ The same study established that using standard cleaning agents eliminated the virus from surfaces.

## COVID-19 TRANSMISSION

As COVID-19 spreads through respiratory droplets, the main components of limiting spread in the workplace are source control and elimination of virus from surfaces. Source control has three main components: 1) preventing employees who are sick from being at work; 2) limiting the spread of droplets from coughing by applying masks to those with a cough and encouraging employees to cough into their elbow and not their hand or into the open air; and 3) encouraging frequent hand washing. The CDC recommends washing hands with soap and water for more than 20 seconds or using a hand sanitizer with at least 60–95% alcohol.^14^ Minimizing the sharing of computers and tools between employees is recommended, as is frequent cleaning of surfaces that employees contact such as door knobs, countertops, keyboards, and phones. This recommendation aims to eliminate the virus’ spread when source control may have failed.^15^ Most of the common cleaning agents registered with the Environmental Protection Agency (EPA) are believed effective in disinfecting surfaces from COVID-19. Alternatively, a mix of five tablespoons household bleach in one gallon of water can be used.^16^ Current information on which cleaning agents can be used on SARS-CoV-2 can be found on the EPA website.^17^

## MITIGATION STRATEGIES FOR PUBLIC SAFETY PERSONNEL

The following proposal applies the above information to mitigating the spread of COVID-19 among public safety personnel working at fire stations or analogous workplaces. It is based on known literature about the nature of SARS-CoV-2. Identifying infected personnel early in order to isolate them from other members should be a key aspect of any mitigation plan. This involves requesting that employees remain at home if they are sick.^14^,^15^ Although this concept may be a sharp departure from cultural norms of being a team player and toughing out illness while at work, it is essential to protect the ability of the public safety workforce to provide adequate staffing to the community. Each agency should identify how absences due to illness will be categorized during this pandemic. For employees who may not have paid sick or vacation days available, finding a mechanism to provide compensation while the employee is unable to work may eliminate part of the incentive an employee may feel to hide his or her symptoms in order to remain at work.

The agency should also consider screening processes in the workplace as an added measure to identify potential COVID-19 cases (or other infections). Screening may include temperature measurement when employees arrive at work and potentially at appropriate intervals during prolonged shifts. It is estimated that 88% of those infected with COVID-19 will develop fever. The CDC defines fever as a temperature of at least 100.4°F/38°C. The screening process may also include questions about new symptoms of respiratory infection such as cough, shortness of breath, myalgias, and sore throat.^4^

Predetermined processes should be employed to manage employees who screen positive. These include determining whether employees who screen positive will be referred to their primary care physician or to the contracted occupational health organization for a medical evaluation, as well as the requirements for return to work. For employees who test positive for COVID-19 during their subsequent medical evaluation, the local public health agency will be an important partner in determining the return-to-work plan based on current national and local criteria. For employees who test negative for COVID-19 or who are believed to have a different etiology for their symptoms, the evaluating medical provider should provide the employee’s return-to-work criteria.

In addition to screening essential personnel at work, agencies may encourage personnel who do not physically need to be in the station to work remotely, as well as limiting visitors to the station. This may further decrease the likelihood that someone with COVID-19 introduces virus into the station. Agencies may also consider a policy that would require employees with a cough to wear surgical masks while on duty. Hand washing or sanitizing should be emphasized both as personnel leave to respond to a call for service, and upon their return to the station. Personnel should also focus on avoiding contact between their hands and face.^8^

## MODIFICATIONS TO SPECIFIC AREAS OF THE STATION

As personnel often cook, eat, work out, and sleep at fire stations, a greater potential for COVID-19 spread may exist in this work environment compared to typical work places. Knowing that COVID-19 spreads via droplet from coughing and hand contact between people, or with surfaces such as door knobs, allows each station to evaluate areas of emphasis to prevent transmission. The agency should identify the surfaces in the station that are contacted routinely. This includes equipment and apparatus parts that are handled, as well as common areas of the fire station or, gym. Scheduled disinfection of these surfaces should be added to the daily routine.

The bathrooms, kitchens, and eating areas should be a particular focus. Although it is not currently believed that COVID-19 is spread primarily via the oral-fecal route, the virus has been isolated in feces and on surfaces throughout the bathroom of an isolated patient.^4^,^13^ With food preparation and eating there may be an increased risk of personnel bringing virus in contact with their mouths. In addition to scheduled surface disinfection or washing surfaces in these areas, an agency may consider requiring hand washing or use of hand sanitizer by personnel both upon entry and exit from those areas. Agencies may consider requiring gloves for food preparation. Steps should also be taken to minimize the sharing of dishes and utensils as much as is feasible.

If the fire station does not currently have single-occupancy bunk rooms, the agency may consider modifying the sleeping quarters so that no more than one person is sleeping in each room. That modification may mitigate the transmission of respiratory droplets between individuals while coughing at night. There should be a dedicated surface wipe down and laundering of linens if applicable during change of shift as well.

Finally, the agency should consider modifying exercise routines for crews. These modifications may include activities that increase distance between individuals and decrease the use of equipment that is touched sequentially. For equipment that is necessary for exercise, implement hand sanitizing and surface disinfection between users.

COVID-19 presents public health challenges to many aspects of society. Its duration and human impact are thus far unknown. During this pandemic, essential work personnel will not be able to work remotely or perform many of the social distancing modifications recommended for other employees. In a pandemic where most of the case clusters occur in households, the fire station may be at particular risk of transmission of COVID-19. This proposal provides potential modifications to station life which can mitigate the spread of COVID-19 among public safety personnel, and preserves their agencies’ ability to fully staff stations to provide for community needs.
